# No negative effects of intra-abdominal bio-logger implantation under general anaesthesia on spatial cognition learning in a hibernator the edible dormouse

**DOI:** 10.1371/journal.pone.0307551

**Published:** 2024-08-28

**Authors:** Tabea Loreen Lammert, Jan Müller, Susana Carolina Ferreira, Ursula Teubenbacher, Jessica Svea Cornils, Gabrielle Stalder, Johanna Painer-Gigler, Thomas Ruf, Claudia Bieber, Friederike Pohlin

**Affiliations:** Department of Wildlife Ecology, University of Veterinary Medicine Vienna, Vienna, Austria; Mustansiriyah University, IRAQ

## Abstract

The effect of hibernation on cognitive capacities of individuals is not fully understood, as studies provide conflicting results. Most studies focus on behavioural observations without taking the physiological state of individuals to account. To mechanistically understand the effect of hibernation on the brain, physiological parameters need to be included. The implantation of bio-loggers can provide insights on i.e. body temperature without further manipulation of the animals. Surgeries and anaesthesia, however, can harm animals’ health and cause cognitive dysfunction, potentially biasing data collected through bio-loggers. We investigated the effects of bio-logger implantation surgery on cognitive performance and learning, controlling for animal and study design characteristics. First, juvenile dormice successfully learned to solve a spatial cognition task using a vertical maze. Distance, transitions, velocity, and duration were measured as indicators for performance. After training, bio-loggers were implanted intra-abdominally under general anaesthesia. Animals were re-tested in the maze two weeks after. We found no effect of bio-logger implantation and surgery on performance. This study is the first to show spatial cognition learning in edible dormice and provides a full description of the peri-anaesthetic management and a protocol for bio-logger implantation surgery in dormice. Importantly, measures were taken to mitigate common anaesthetic complications that could lead to post-operative cognitive dysfunction and influence animal behaviour. By pairing physiological measurements through bio-logger implantation with behaviour and cognition measurements, future research will significantly advance the understanding on mechanisms of learning and behaviour.

## Introduction

Manoeuvring through different environments and remembering locations like feeding sites is a cognitive ability widespread in the animal kingdom and is essential for fitness [1–3]. Thus, intensive research has been done to unravel and compare learning strategies and fundamental mechanisms in this context (for review see [4, 5]). Navigating complex environments has led to the emergence of diverse traits and behaviour strategies(for review see [6]). Depending on habitat complexity, learning and orientation can be challenging, thus leading to the implementation of different approaches and cues [7]. Spatial cognition is mostly researched in rodents or primates (e.g. [3, 4, 8, 9]) and a variety of setups such as Barnes Mazes were developed to investigate learning strategies under laboratory and natural conditions (for review see [1, 10]. However, less is known about arboreal or even nocturnal and arboreal species because canopies are a highly complex and dynamic environment [11]. Thus, it is challenging to situate animals and their exact movements within the detailed canopy network [12, 13].

In our study we focused on the edible dormouse (*Glis glis*), a nocturnal and arboreal rodent (e.g. [14, 15] that can hibernate up to 11 months [16]. In edible dormice, explorative behaviour has been studied (e.g. [17, 18]) but insights on spatial cognition and learning are still lacking. For species like edible dormice that go into hibernation, an extreme physiological state to survive unfavourable environmental conditions and avoid predation [19, 20], it is hypothesized that brain energy requirements and size are under negative selection which may lead to cognitive impairment compared to non-hibernating species [21–23]. Consequently, hibernation could result in a drawback for brain function and may induce an impaired ability to cope with novel or fluctuating environmental conditions. Hibernation is characterized by torpor bouts that can last days up to weeks depending on the species [24]. Because of reduced brain activity during torpor and a possible decrease in brain size, several studies looked at the effect of hibernation on learning with variable outcomes (e.g. [25–27]. Most of these studies focussed on behavioural observation and did not include physiological parameters (i.e. hibernation patterns). But to understand physiological performance with different environmental challenges it is crucial to understand species adaption to environmental changes[28].

Physiological parameters can be recorded using bio-logging devices that are either externally attached or implanted into the animal [29]. Intra-abdominal implantation of bio-loggers is advantageous for long term studies, but these procedures are performed under general anaesthesia and post-operative care is required to ensure animals can return to their routine behaviour as soon as possible [29, 30]. It is known that surgeries can negatively impact animal behaviour [31, 32] and are always a risk to the animals’ health in general, which is why the benefits of bio-logger usage need to be balanced against the cost of bio-logger implantation [33]. Several studies report cognitive deficits after general anaesthesia (for review see [34, 35]) and the possibility to influence memory capacity by impairing short-and long-term memory [36, 37] and causing disorders in spatial cognition [38]. It is currently unclear whether surgical implantation of intra-abdominal bio-loggers under general anaesthesia influences spatial cognition learning in edible dormice.

This study aimed at establishing baseline references for the effects of experimental setup (trials, bio-logger implantation under general anaesthesia and time of experiment) and individual characteristics (body mass, age, sex and maternal effects) on cognitive performance in juvenile edible dormice prior to their first hibernation. We hypothesized that 1) juvenile dormice can solve a vertical maze prior their first hibernation, increase performance and retain memory with ongoing training 2) that intra-abdominal bio-logger implantation under general anaesthesia does not negatively affect the maze-performance and memory retention of the animals.

## Materials & methods

### Ethics statement

The experimental procedures were approved by the Ethics and Animal Welfare Committee of the University of Veterinary Medicine, Vienna in accordance with the University’s guidelines for Good Scientific Practice and authorized by the Austrian Federal Ministry of Education, Science and Research (ref BMBWF 2020–0.193.178, ref BMBWF 2021–0.370.463) in accordance with current legislation.

### Animals

51 dormice (*Glis glis*), born in a breeding colony at the Institute of Wildlife Ecology in Vienna, Austria, 2021 and 2022, were used for this study. Dormice were removed from their mother at 21 days of age (directly after opening their eyes) and hand-raised afterwards (for details see S1 File). In 2021 we took 31 pups (14m, 17f) from 12 different litters (mothers’ age 4–6 years) and in 2022 we took 20 pups (14m, 6 f) from 5 litters (mothers’ age 4–9 years). After hand raising, animals were kept in groups of max. 10 animals with mixed gender in outdoor enclosures (2x1x1 m). Outdoor enclosures did not allow to access underground to prevent them from going into early hibernation during experiments. Nest boxes and branches were available, rodent chow (Ssniff Gerbil, Ssniff, Soest, Germany) and water was provided *ad libitum*.

### Training and testing

**Fig 1:**
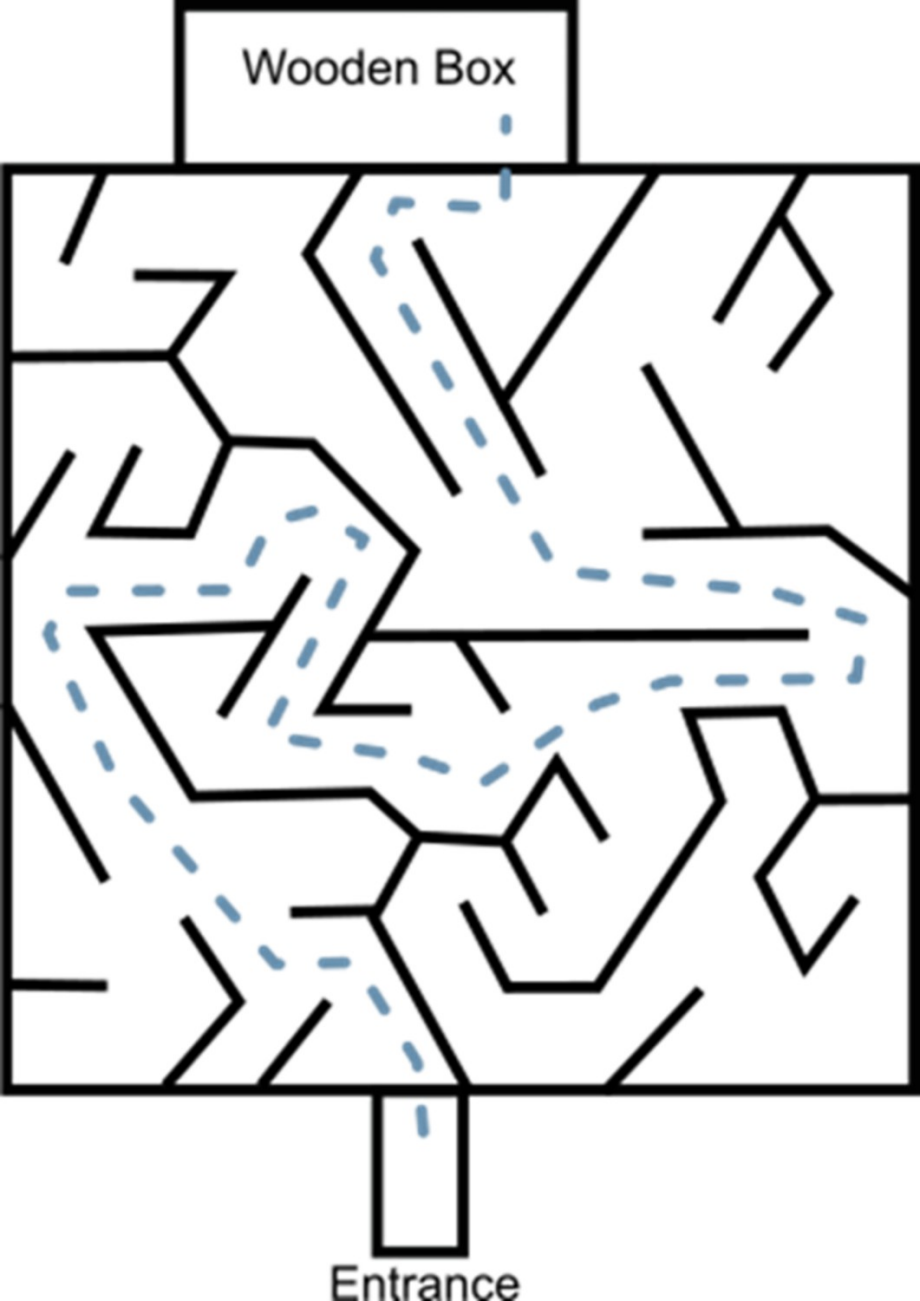
Schematic figure of the vertical maze 1 m x 1 m that was used as the spatial cognition task to be solved by juvenile edible dormice n = 51. The dashed line represents the shortest and direct path (261 cm). Performance was measured as distance, transitions, velocity and duration.

At 5 weeks of age, animals were habitua to a wooden box in which pups were rewarded with food (Bio Hipp Hippis, Hipp, Sachseln, Switzerland) when entering. The wooden box was later used as the destination box for the spatial cognition task (vertical maze, Fig 1).

At 6 weeks of age, pups were challenged in the vertical maze for the first time. The training schedule, prior to surgical implantation of an intra-abdominal bio-logger, lasted for 14 days, whereby animals were trained in a maze every second day (= 7 sessions). Animals were weighed prior to every session to the nearest of 1g (Sartorius EA35DE-I, Sartorius AG, Göttingen, Germany). To cover for dormice activity patterns, experiments were conducted 3 hours before sunset when dormice became more active (personal observation from our outdoor enclosures). Test-order of the animals was pseudorandomized. Training started with a maximum of 3 trials in the first session with no breaks between trials. A trial was considered finished when the animal travelled from the entrance tube to the reward box. After every trial, the maze was cleaned using 70% Isopropanol to eliminate odour cues. Animals were set at start after finishing the trial successfully and having received the reward. The number of trials were increased by one trial with every session to a maximum of 7 trials on the last trainings day. Due to animal welfare-reasons, one session was limited to 30 min. Therefore, number of trials did not exceed 3 in the first session because animals required more time to solve the task in a new environment. Experiments were performed under red light and recorded using a camera (Bosch Dinion IP 5000 HD, Bosch, Stuttgart, Germany). After bio-logger implantation surgery dormice rested for 14 days and were tested once again in the vertical maze using the scheme as described above. The only difference in this session after bio-logger implantation surgery was that the number of trials per animal was reduced from a maximum of 7 to 5 trials, because animals were found to be less responsive to the food reward. We measured performance on the first session (novel environment, maximum of 3 trials/individuum), and the sessions before anaesthesia (maximum of 7 trials/individuum) and after anaesthesia (maximum of 5 trials/individuum). Performance was measured as the distance in cm per trial dormice travelled in the maze and counted the transitions, i.e., deviations from the shortest path. One transition was counted when the animal entered the maze, leading to a minimum of 1 transition in general. Additionally, we measured the mean velocity of the animals (cm/s) per trial and recorded the duration in s to complete the trial.

### Loggers and implantation

At two months of age, all dormice were implanted with wax-coated home-made bio-loggers (after [39], logger size: 16x12x8 mm, weight 2.5 g, capacity: ~105,000 data points, 10-minute interval of recording). The loggers were implanted to record body temperature and activity especially during hibernation in dormice. These data are not part of the present study and will be published separately.

Implantations were carried out by veterinarians in a fully equipped surgery room at our Institute. Anaesthesia for intra-abdominal bio-logger implantations was induced by a subcutaneous (SC) injection of 50 mg/kg ketamine (Ketamidor® 10%, Richter Pharma Wels, Austria) and 8 mg/kg xylazine (Rompun® 2%, Bayer, Leverkusen, Germany) into a loose skin fold over the flank of the animals. After the injection, the dormice were placed into a fabric bag and left unstimulated for 10 minutes to provide a calm and quiet environment that allowed for the drugs to take effect. Once anaesthetized, animals were positioned in dorsal recumbency on a surgical table specifically designed for mice and rats warmed to 40°C (Combi-vet® Surgery table System 3, Rothacher Medical GmbH, Heitenried, Switzerland). This table included a facemask through which anaesthesia was maintained with a mean of 1 ± 0.7% isoflurane (Vetflurane® [1000 mg/g], Virbac Austria GmbH, Vienna, Austria) in 100% oxygen. Eye ointment (Vit-A-Vision®, Omnivision GmbH, Puchheim, Germany) was applied, 2 mg/kg meloxicam (Metacam® [2 mg/ml], Boehringer Ingelheim Vetmedica GmbH, Ingelheim, Germany) was injected SC, and a SC fluid bolus of 10 ml/kg Ringer lactate solution (Ringer-Lactat®, Braun, Melsungen, Germany) administered. The abdominal skin of the animals was clipped with an electric shaver and aseptically prepared for surgery. To avoid mechanical irritation of the skin, we did not additionally treat the area with a blade razor. Laparotomy was performed through a 1cm abdominal midline incision, and the bio-logger placed free-floating into the abdominal cavity. The incision was then closed with simple interrupted intramuscular sutures (Surgicryl®, PGA, USP 3–0, 75cm, TP19), a splash block with 1 mg/kg lidocaine (Xylanaest® purum 1%, Gebro Pharma GmbH, Fieberbrunn, Austria) was applied to provide topical analgesia, and the skin then closed with continuous intradermal sutures (PGA PLUS®, 1,5 metric, USP 4–0, 70cm, 3/8, RC19). The latter was necessary to avoid that animals gnaw at reachable knots. Standard anaesthesia monitoring was performed during surgery and included: pulse rate (PR) and peripheral oxygen haemoglobin saturation (SpO2) recorded every 5 minutes by a pulse oximeter attached to a hind paw (Root™ Radical-7, Masimo Corporation, Irvine, CA, USA); respiratory rate measured every 5–10 minutes by counting the number of thoracic excursions; and body temperature measured at the beginning and end of surgery using a rectal thermometer (HS Digital Veterinary Thermometer, Henry Schein Animal Health, Wien, Austria). Sufficient intraoperative depth of anaesthesia was ensured by a negative pedal withdrawal response and negative response to surgical stimulus.

### Post-operative care

For recovery of anaesthesia, each animal was placed in an individual type 2 rodent cage. Body temperature was maintained by an adjustable heating pad placed below the cage and flow-by oxygen (0.5 L/min) provided until the animals were fully awake (~30 min). For post-operative care, animals were placed in individual inside-enclosures (59 cm x 49 cm x 41 cm) and provided with shelter (small wooden box, 15 cm x 16 cm x 18 cm), food (Ssniff Gerbil, Ssniff, Soest, Germany, apples, and sunflower seeds), and water *ad libitum*, for 5–7 days after the surgeries. For 3 days, each animal was administered 2 mg/kg meloxicam orally. Behaviour and wound healing were monitored and assessed daily using a previously established score [40]. Animals returned to their original enclosures, once completely healed, after surgery.

### Statistics

Since all animals were repeatedly tested, each individual served as its own control group in our study.

Videos were tracked using EthoVision®XT (Noldus, WashingtonDC, USA). Performance parameters were evaluated with the tracking software.

EthoVision®XT allows to calculate the travelled distance in cm of every individual. It is calculated as

$$DM_{n}=\sqrt{{\left(X_{n}-X_{n-1}\right)}^{2}+{\left(Y_{n}-Y_{n-1}\right)}^{2}}$$

With DM_n_ as distance moved from sample n-1 to sample n, X_n-1_, Y_n-1_ = X, Y coordinates of the center point at sample n-1 to sample n, X_n-1_, Y_n-1_ = X, Y coordinates of the center point. Shortest distance was 261 cm. We assumed that reduced distance shows learning of the animal.

Transitions are counted as number of times an animal visits a defined zone. We defined the shortest path as a zone to count how many times animals left the shortest path. When animals entered the maze for the first time, 1 transition was counted. We assumed the task to be learned when animals showed few or no errors (less transitions from shortest path).

Velocity of animals is obtained by dividing Distance moved by the time difference between a sample and previous one:

$$V_{n}=\frac{{DM}_{n}}{t_{n}-t_{n-1}}$$

V_n_ = velocity at sample n (expressed in the unit of the defined experiment settings) and DM_n_ = Distance moved at sample n. We assumed that velocity gives a measure with respect to general activity.

Duration was recorded in seconds by the software. We measured the required time from entering the maze to reaching the nest box. Duration gives information of how much time animals needed to solve the task. We assumed improvement of performance when duration per trial decreased.

All statistical analyses were performed using R version 4.3.2 (R Core Team, 2022).

To investigate the effects of bio-logger implantation and general anaesthesia on cognitive performance, we applied a general mixed effect models with the family “negative binominal” using the “glmer.nb” function of the “lme4” R package [41] for each performance variable (distance, transitions, velocity and duration). As fixed effect parameters we included session (first session; pre-surgery; post- surgery), trial, sex, age, body mass and time of the day at the start of each session. Start time was calculated as $\sin(\frac{\left(Time\;of\;the\;day\right)}{\left(24*2*\pi \right)})$. To account for different learning curves within each session, we include an interaction between session and trial. Individual ID was included as random effect. We applied log-likelihood ratio tests (LRT) to evaluate the significance of the marginal contribution of each predictor variable to the full model by removing each predictor and comparing against the full model [42]. Goodness of fit for each model was accessed by the variance explained by the fixed effects (marginal R square values) and fixed and random effects (conditional R square values) [43], calculated with the function “r.squaredGLMM”, R Package “MuMIn” [44] using the trigamma method. Additionally, global goodness of fit was accessed as LRT against the intercept-only model. We tested differences between sessions with pairwise comparison of estimated means pairwise post hoc comparisons using the R Package “emmeans” [45]. Resulting marginal mean estimates were averaged over sex levels and presented on log scale and resulting p-values of pairwise post hoc comparisions were adjusted with the Benjamin-Hochberg method. If not stated otherwise, all mean values were given ± standard error of the mean (s.e.m.). We imputed 36 measurements of body mass (4.68%) that were missing with the function mice, method “cart”, of the R package mice [46]. Plots were generated using “ggplot2” [47].

## Results

### Dormice in a novel environment: Increased performance with trials

**Fig 2:**
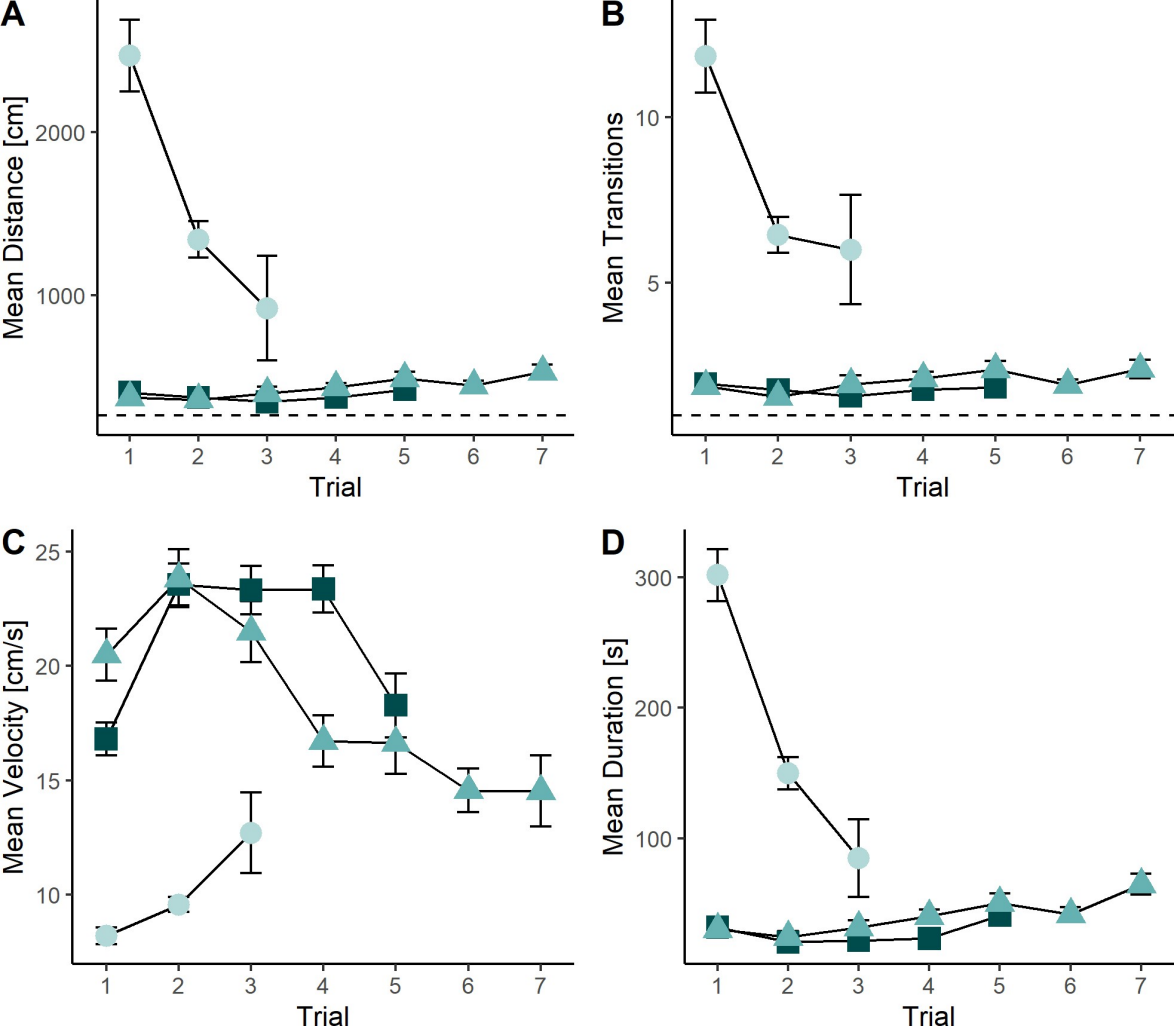
Mean performance in maze over different sessions, n = 51, maximum time limit 30 min per animal. First Training corresponds to the first training session where animals were unexperienced, maximum 3 Trials. Pre-Surgery corresponds to the last training session 1 day prior to surgery and 13 days after the first trainings session, maximum 7 Trials. Post-surgery corresponds to the first test session after surgery and 14 days of recovery, maximum 5 Trials A) Mean distance in cm covered in maze over trials. Shortest distance to pass through the maze: 261 cm B) Mean transitions (counts) from direct path per trial, minimum transition counted: 1 (i.e. entering the maze-frame) C) mean velocity in cm/s D) mean duration per trial in seconds.

The shortest path through the maze was 261 cm. The interaction between session and trial on each performance measure (Distance, Transitions, Velocity and Duration) was significant (Table 1). When challenged in the maze for the first time, dormice travelled great distances (trial 1: mean 2471 cm ± SD, 404:6820 cm, Fig 2A). Distance reduced in the second trial (mean 1342 cm ± SD, 314:3710 cm) and continued to improve on the last trial (trial 3: mean 919 cm ± SD, 307:2070 cm). Large distances were accompanied by high transitions from the shortest path. Transitions on the first trial were highest (mean 11.8 ± SD, 2:33, Fig 2B) and decreased in the following trials (trial 2: mean 6.45 ± SD, 1:17; trial 3: mean 6 ± SD, 3:12). Dormice were the slowest in trial 1 (velocity mean 8.20 cm/s ± SD, 4.19:14.4 cm/s, Fig 2C) and the velocity increased over trials 2 and 3 (trial 2: mean 9.57 cm/s ± SD, 5.77:16.4 cm/s, trial 3: mean 12.7 ± SD, 9.13:12.7 cm/s). Increased distance, high transitions and slow velocity also led to increased duration for the first trial (mean 301 s ± SD, 22:194 s). Duration decreased over the next trials (trial 2: mean 150 s ± SD, 21.4:384 s; trial 3: mean 85 s ± SD, 22:194 s, Fig 2D). Performance improved in the pre- and post-surgery sessions. Within these sessions performance remained stable or reduced for each consecutive trial (Fig 2). Additionally, there was a significant positive effect of start time on velocity and duration, and a sex effect on transitions, with males having fewer transitions than females (Table 1). However, the latter is weak and was only observed in one tested variable.

**Table 1:** Animal characteristics and experimental setup effects on performance as estimated by generalized mixed-effect models.

	Estimate	s.d.	Z	p-value	χ2	df	p-value
**Distance**							
Intercept	6.1963	0.1828	33.8983	<0.0001	-	-	-
Session [First]	2.3531	0.1386	16.9809	<0.0001	417.13	2	**<0.0001**
Session day [post-surgery]	-0.1724	0.0827	-2.0847	0.0371			
Bodymass	0.0015	0.0015	0.9940	0.3201	0.9961	1	0.3183
Sex [male]	-0.0791	0.0466	-1.6978	0.0896	2.8437	1	0.0917
Age	-0.0039	0.0021	-1.8607	0.0628	3.2707	1	0.07053
Start time	-17.2605	13.0630	-1.3213	0.1864	0.0591	1	0.8080
Trial	0.0074	0.0152	0.4856	0.6273	86.3116	2	**<0.0001**
Session [First]* Trial	-0.5799	0.0701	-8.2750	<0.0001	76.9110	1	**<0.0001**
Session [Last]* Trial	0.0505	0.0191	2.6480	0.0081			
**Transitions**							
Intercept	1.0849	0.3489	3.1098	0.0012	-	-	-
Session [First]	2.2671	0.1929	11.7474	<0.0001	225.7560	2	**<0.0001**
Session day [post-surgery]	-0.1381	0.1579	-0.8748	0.3817			
Bodymass	-0.0003	0.0025	-0.1339	0.8935	0.01794	1	0.8935
Sex [male]	-0.1400	0.0685	-2.0435	0.0410	4.1295	1	**0.0421**
Age	-0.0018	0.0036	-0.5029	0.6136	0.2376	1	0.2376
Start time	-51.7063	37.3711	-1.3836	0.1665	0.2410	1	0.6235
Trial	-0.0129	0.0309	-0.4185	0.6756	42.8692	1	**<0.0001**
Session [First]* Trial	-0.4971	0.0862	-5.7650	<0.0001	42.8517	1	**<0.0001**
Session [Last]* Trial	0.0577	0.0377	1.5307	0.1259			
**Velocity**							
Intercept	2.3122	0.2188	10.5684	<0.0001	-	-	-
Session [First]	-1.1201	0.1602	-6.9932	<0.0001	81.0611	2	**<0.0001**
Session day [post-surgery]	0.1982	0.0868	2.2829	0.0224			
Bodymass	-0.0022	0.0016	-1.3658	0.1720	1.8566	1	0.1730
Sex [male]	-0.1064	0.0607	-1.7528	0.0796	2.9802	1	0.0843
Age	0.0022	0.0023	0.9538	0.3412	0.8651	1	0.3523
Start time	191.2260	22.0993	8.6530	<0.0001	5.7613	1	**0.0164**
Trial	0.0089	0.0160	0.5523	0.5807	58.7225	2	**<0.0001**
Session [First]* Trial	0.1874	0.0815	2.3004	0.0214	32.2209	1	**<0.0001**
Session [Last]* Trial	-0.0958	0.0199	-4.8238	<0.0001			
**Duration**							
Intercept	4.2279	0.3511	12.0324	<0.0001	-	-	-
Session [First]	3.1616	0.2382	13.2718	<0.0001	267.06222	2	**<0.0001**
Session day [post-surgery]	-0.23621	0.1440	-1.6406	0.1009			
Bodymass	0.0017	0.0027	0.6158	0.5380	0.37979	1	0.5377
Sex [male]	0.0089	0.0986	0.0905	0.9279	0.0081	1	0.9285
Age	-0.0075	0.0040	-1.8727	0.0611	3.3169	1	0.0686
Start time	-168.7625	25.0262	-6.7434	0.0001	267.0622	1	**<0.0001**
Trial	0.0547	0.0248	2.2083	0.0272	96.1502	3	**<0.0001**
Session [First]* Trial	-0.7673	0.1177	-6.5190	<0.00001	55.3022	2	**<0.0001**
Session [Last]* Trial	0.1011	0.0317	3.1925	0.0014			

769 observations from 51 individuals. Performance is measured as distance (cm), transitions (units), velocity (units), duration (s). The parameter estimates are reported with the corresponding standard error (s.d.), Z-statistics and respective p-values (Wald test). Also shown are the tests for the significance of each parameter using log-likelihood ration test (χ2) with the corresponding degrees of freedom (df) and p-value. Post-surgery session is the reference value for the session predictor and female is the reference for the sex predictor. Significant effects are printed in bold.

### No effect of general anaesthesia and implantation on performance

**Fig 3:**
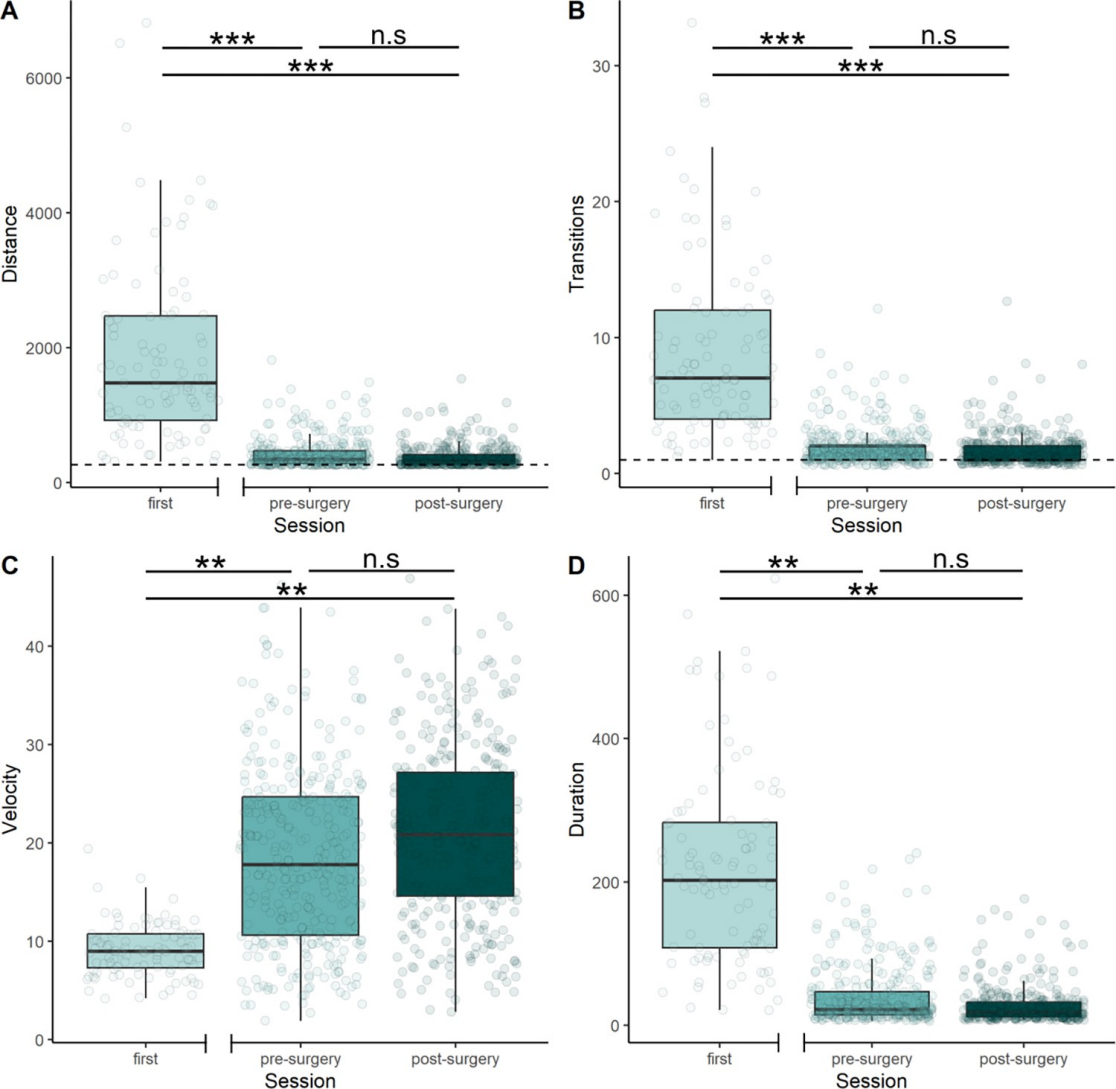
Performance in the maze in different sessions: First time, pre-surgery and post-surgery, n = 51. First session corresponds to the first training session where animals were unexperienced. Pre-Surgery corresponds to the last training session 1 day prior to surgery and 13 days after the first trainings session. Post-surgery corresponds to the first test session after surgery and 14 days of recovery A) Distance in cm covered in maze, direct path = 261cm B) Transitions from the direct path C) Velocity of animals in cm/s D) Duration in seconds to finish trials. Differences between first vs. pre-surgery session and first vs. post-surgery session were significant for every performance variable. Significant differences at ≤0.01, ≤0.001, ≤0.0001, levels are highlighted using **,*** and **** respectively.

We investigated the effect of bio-logger implantation, with linear mixed-effect models (LME), which allow controlling for animal and set-up related factors on the performance variables. Overall, the proportion of variance explained by the fixed-effect predictors is large (marginal R2 for distance: 0.585, transition: 0.330, velocity: 0.304 and duration: 0.43), whereas individual ID explained a moderate proportion of the variance as seen by the conditional R2 which includes the variance explained by both fixed and random effects (distance: 0.619, transitions: 0.359, velocity: 0.417 and duration: 0.510). There was a significant difference in performance between first session and pre-surgery session and between first session and post-surgery session (Table 2, Fig 3). No difference was detected between pre- and post-surgery session.

**Table 2:** Turkey’s post hoc pairwise comparisons for each level of session.

	First vs. pre-surgery	First vs. post-surgery	Post- vs. pre surgery
**Distance**			
Estimate difference	0.5285	0.5159	0.0125
Z-ratio	4.396	3.840	0.230
p-value	**<0.0001**	**0.0002**	0.8178
**Transitions**			
Estimate difference	0.6479	0.6924	-0.0445
Z-ratio	4.200	3.742	-0,452
p-value	**0.0001**	**0.0003**	0.6515
**Velocity**			
Estimate difference	-0.421	-0.526	0.105
Z-ratio	-3.060	-3.424	1.811
p-value	**0.0033**	**0.0019**	0.0702
**Duration**			
Estimate difference	0.647	0.731	-0.084
Z-ratio	3.189	3.168	-0.854
p-value	**0.0023**	**0.0023**	0.3929

P-Value adjustments with Benjamin-Hochberg method. Estimates are on the log scale and results are averaged over levels of sex. Significant effects are printed in bold.

### Stable physiological parameters under general anaesthesia

**Fig 4:**
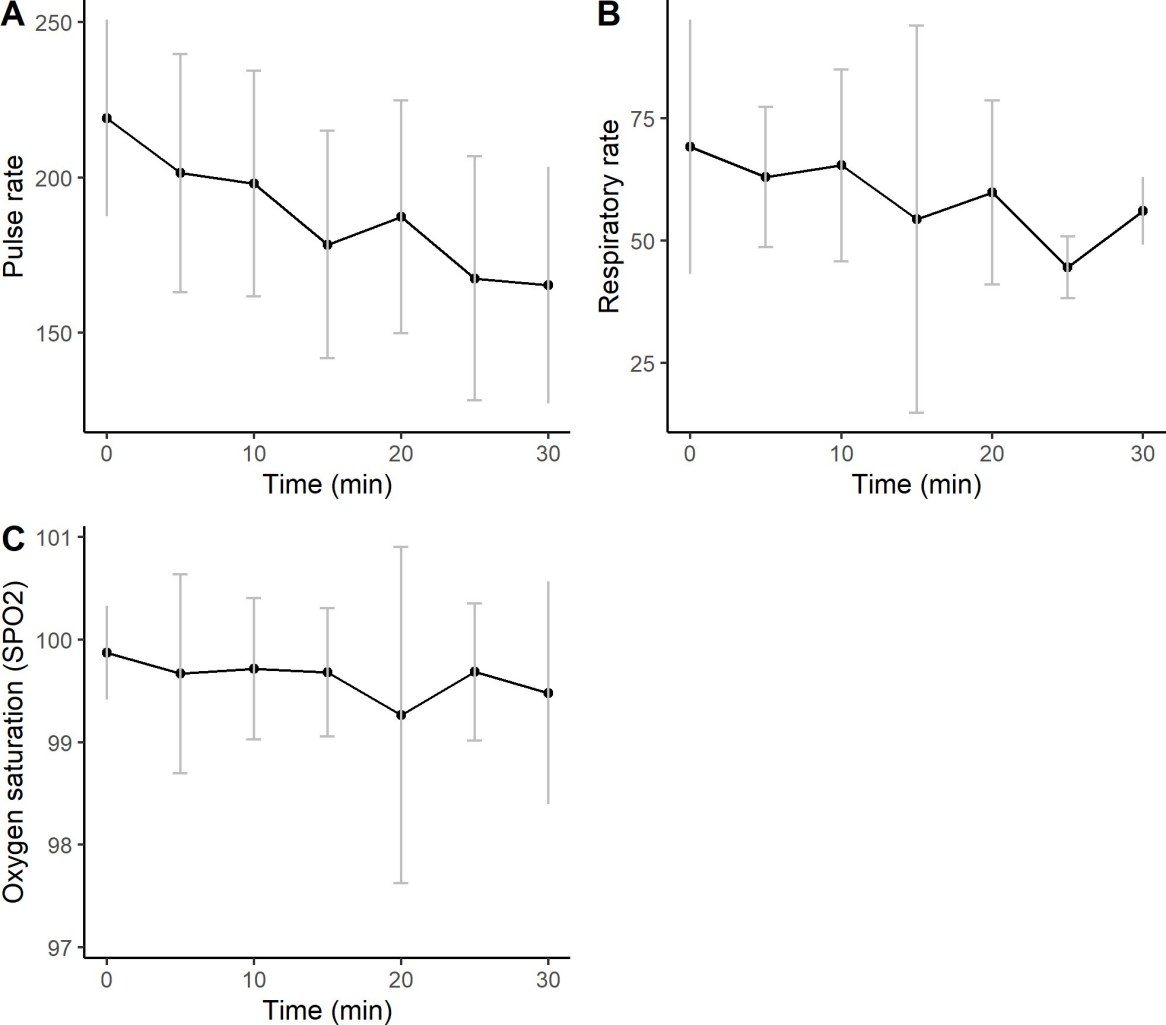
Monitoring of physiological parameters during anaesthesia a) Pulse rate (PR) in beats per minute (Time) b) Respiratory rate (RR) in breaths per minute (Time) c) Oxygen saturation (SpO2) in % over time.

General anaesthesia was performed with all participating dormice. The mean ± SD surgery- and anaesthesia times were 22.5 ± 5 and 30.6 ± 6.5 minutes, respectively. Mean ± SD overall pulse rate under anaesthesia was 188 ± 40 beats per minute (bepm), decreasing gradually over time from 220 ± 32 bepm at the beginning to 168 ± 38 bepm at the end of anaesthesia (Fig 4A). Overall mean ± SD respiratory rate was 63 ± 22 breaths per minute (brpm), decreasing from 71 ± 26 brpm at the beginning to 56 ± 7 brpm at the end of anaesthesia (Fig 4B). Overall mean ± SD SpO2 was 99.5 ± 1% and never dropped below 98% in any anaesthetized animal (Fig 4C). Mean body temperatures were 36.7± 0.8°C before and 36.4 ± 0.8°C after anaesthesia.

## Discussion

For the first time, spatial cognition learning could be shown in edible dormice. Juvenile dormice solved the vertical maze and retained memory during their active season. Animals showed a steep learning curve within the first session. Initially, dormice explored the maze intensively, shown by long distances travelled, high numbers of transitions (leaving the correct path), long durations, and low velocity (speed) to finish the trials. Already on the first trial of the first session, juvenile dormice showed enhanced performance demonstrating their potential to quickly learn and adapt to new environments. This finding aligns with previous reports about learning in juvenile rodents [48–50] and is also supported by results from a spatial cognition study in *Ctenomys talarum*, which also showed improvement in a maze setup already during the first trainings session [51]. In structurally complex environments, it is expected that spatial learning abilities of animals are under selective pressure and are beneficial to build representations of the environment to improve navigation and orientation [8, 51]. All dormice solved the vertical maze, showing that these rodents can learn a cognitive task prior their first hibernation in a setup that simulates the challenges in their natural, arboreal environment. The usage of vertical mazes in which animals must climb upwards to reach food and shelter, is relatively uncommon. One study investigated spatial orientation in free ranging fox squirrels using a vertical maze [52]. Another study tested rats in a three-dimensional maze, indicating the animals’ ability to manoeuvre through different dimensions [53]. Our study is the first to show spatial cognition learning in a vertical maze in edible dormice, providing a baseline for future studies on memory retention of the learned task and hibernation effects.

To measure physiological parameters during hibernation, animals need to be equipped with bio-loggers. While external logger attachment is less invasive, it can decrease fitness, hinder movements [54] and even cause neck lesions [55, 56]. Dormice show fast increased fattening prior hibernation (e.g. [20, 57]), therefore collars are not feasible for this species, and we opted for surgical bio-logger implantation. The biggest drawback of this procedure is that is invasive and carries a small risk of chronic inflammation or even device rejection [58]. In Adelié penguins for example, birds that were implanted with intra-abdominal bio-loggers showed increased foraging periods and longer recovery on the water surface compared to birds with externally attached loggers [31]. The authors hypothesized that increased energetic cost associated with tissue repair as well as an increased intra-abdominal pressure caused these behavioural alterations. Another important factor to be considered is the general anaesthesia and post-operative care associated with the invasiveness of the surgical implantation [29]. Rodents in particular have a high risk of anaesthetic related deaths (i.e. for rats the reported anaesthetic mortality rate is 2.01%) [59]. Fluid loss (hypovolemia), hypothermia, and respiratory depression (leading to hypoxaemia) represent the main anaesthetic complications documented in small mammals [60]. While cognitive dysfunction is a common complication immediately after surgery, it can also cause impaired memory and learning that last for longer periods of time [61, 62]. In our study, all animals survived general anaesthesia and intra-abdominal bio-logger implantation with no observed behavioural alterations or wound healing complications after surgery. Our results show that performance is similar before and after a 14-day resting period after surgery. This indicates that these procedures did not negatively affect memory and cognition in our dormice.

State-of-the-art peri-anaesthetic management and close monitoring of physiological parameters allowed us to provide optimal animal health during surgery minimizing the risk of postoperative cognitive dysfunction (POCD). POCD is associated with increased age, intraoperative anaesthetic complications (i.e., hypovolemia, hypoxaemia, hypothermia), postoperative infections, duration of anaesthesia, and repetitive surgeries [63]. In most studies in rodents, cognitive impairment is also more likely to occur in older animals compared to younger ones [38, 64]. In our study, we only implanted juvenile dormice, thus leading to the possibility, that results in adult dormice might differ and need to be considered in future research. Multiple surgeries did not seem to have a negative impact on spatial memory in adult mice [65], but neonatal mice receiving multiple doses of ketamine-xylazine anaesthesia showed deficits in novel object recognition, sociability, preference for social novelty and contextual fear response [66]. Single dose of ketamine-xylazine anaesthesia, on the contrary, had no effect on these behaviours, which agrees with the findings of our study where no POCD was identified after a single anaesthetic event using the same drug combination in juvenile edible dormice. Hypoxaemia and intraoperative hypothermia have been associated with impaired spatial learning and POCD in rats, respectively [67, 68]. We mitigated these complications by supplementing oxygen during the entire perianaesthetic period and providing active warming to the animals. Fluid loss was replaced by providing SC fluids and surgery times kept to a minimum. To decrease the risk of postoperative infections, surgeries were carried using aseptic techniques and analgesia provided by administering non-steroidal anti-inflammatory drugs (NSAID). The stable anaesthesia monitoring data, short surgery- and anaesthesia times, as well as the fact that our animals did not show marked postoperative infections prove that these anaesthetic management techniques were implemented successfully to minimize the risk of POCD. In fact, factors that have been shown to decrease the risk of POCD in humans include the use of NSAID, ketamine, and alpha-2-antagonist; drugs that were included in our anaesthesia and analgesia protocol [63].

Interestingly, there was an increase in the variance of velocity of the animals in the sessions pre-and post-surgery. One possible explanation is the need to forage to prepare for hibernation. Towards the end of the active season, dormice need to increase their fat reserves to be able to go into hibernation (e.g., [69, 70]). During this time, it can be useful to benefit from all available food sources, leading to an extended need to forage. Variation in velocity could also be caused by differences in their motivation for movement, possibly shaped by intrinsic (their personality) and environmental or social context factors [71]. However, our study design may not be suitable to assess personality and consequently lack e.g. reliability [72]. Further research and a fitting experimental design are required to make reliable statements on personality differences in edible dormice. Aside from the session, start time appeared to have influenced the velocity and duration of the animals, with better performances during later hours. Dormice are a nocturnal species, consequently, their activity levels are the highest during the night [73]. We thus conducted each session in the evening in the absence of sunlight. It is not unlikely that the animals’ velocity is lower at the beginning of their active phase and animals become faster as they gain activity.

## Conclusion

We found no negative impact of general anaesthesia and bio-logger implantation on health, memory and behaviour in edible dormice. Our results can help pave the way for future studies on learning and behaviour that will also cover for physiological parameters (i.e. body temperature, activity, heart rate), an aspect which seems, in our view, underrepresented in behavioural studies. Indeed, implantation of bio-loggers can provide important insights by linking behaviour with corresponding physiological parameters. Especially for hibernating species, where the impact of hibernation on the brain is not yet fully understood, more research is required that not only observes behaviour from the outside but also covers physiological parameters. Additionally, physiological data can help improve the understanding of animal health and adaption to the changing and fluctuating environment, especially in context of global warming and thus help to find and support conservation strategies. Our developed protocols and here carefully described techniques for implantation can be seen as a successful standard, which can be adapted for other species as well.

## Supporting information

S1 File Handraising and keeping conditions of edible dormice. Animals were taken from our breeding colony at the Research Institute of Wildlife Ecology, University of Veterinary Medicine Vienna (for details see [57]). For our experiment it was mandatory that the dormice were not afraid of humans. Therefore, we hand raised animals (in 2021 n = 30, 13 males, 17 females and 2022 n = 20, 14 males, 6 females). Pups were removed from their mother when their eyes started to open (~21 days as described in [74]). Litters were born between June to July in both years. After removal from their natal nest, pups were held in a small transport box (40x26x23 cm, AniOne, Krefeld, Germany) and fed every 2 hours with human milk formula (Bepa expert HA PRE, Nestlé, Vevey, Switzerland). Powder of intestine bacteria was added (Enteroferment, Richterpharma, Wells, Germany) to prevent diarrhoea. At 24 days of age, pups were fed with milk every 3 hours. Fruit mesh (Bio Hipp Hippis, Hipp, Sachseln, Switzerland) and porridge (Milchbrei, Milupa, Frankfurt, Germany) was offered. At 25 days, sunflower seeds and apples were introduced and milk was reduced. To monitor growth, animals were weighted daily (balance 3pm Dipse TP 500, Oldenburg, Germany) to the nearest of 0.1 g. As pups became more mobile, they were transferred to birdcages (66x66x155 cm, VidaXL, Venlo, Netherlands) and were offered branches, platforms and nest boxes. At 27 days, pups were marked individually with transponders (ISO-Transponder, Tierchip Dasmann, Tecklenburger Land, Germany). At 30 days of age, pups were transferred to outside enclosures (2x1x1 m) with fenced floor, where they were kept in groups of 10 individuals. Nest boxes and branches were available, rodent chow (Ssniff Gerbil, Ssniff, Soest, Germany) and water was provided *ad libitum*.
(DOCX)

S1 Data (ZIP)
