# Supplementary material for: No negative effects of intra-abdominal bio-logger implantation under general anaesthesia on spatial cognition learning in a hibernator the edible dormouse
Source: PLoS One. 2024 Aug 28;19(8):e0307551. doi: 10.1371/journal.pone.0307551 (PMC11356448; doi:10.1371/journal.pone.0307551)
Supplement: S1 File — Animals were taken from our breeding colony at the Research Institute of Wildlife Ecology, University of Veterinary Medicine Vienna (for details see [57]). For our experiment it was mandatory that the dormice were not afraid of humans. Therefore, we hand raised animals (in 2021 n = 30, 13 males, 17 females and 2022 n = 20, 14 males, 6 females). Pups were removed from their mother when their eyes started to open (~21 days as described in [74]). Litters were born between June to July in both years. After removal from their natal nest, pups were held in a small transport box (40x26x23 cm, AniOne, Krefeld, Germany) and fed every 2 hours with human milk formula (Bepa expert HA PRE, Nestlé, Vevey, Switzerland). Powder of intestine bacteria was added (Enteroferment, Richterpharma, Wells, Germany) to prevent diarrhoea. At 24 days of age, pups were fed with milk every 3 hours. Fruit mesh (Bio Hipp Hippis, Hipp, Sachseln, Switzerland) and porridge (Milchbrei, Milupa, Frankfurt, Germany) was offered. At 25 days, sunflower seeds and apples were introduced and milk was reduced. To monitor growth, animals were weighted daily (balance 3pm Dipse TP 500, Oldenburg, Germany) to the nearest of 0.1 g. As pups became more mobile, they were transferred to birdcages (66x66x155 cm, VidaXL, Venlo, Netherlands) and were offered branches, platforms and nest boxes. At 27 days, pups were marked individually with transponders (ISO-Transponder, Tierchip Dasmann, Tecklenburger Land, Germany). At 30 days of age, pups were transferred to outside enclosures (2x1x1 m) with fenced floor, where they were kept in groups of 10 individuals. Nest boxes and branches were available, rodent chow (Ssniff Gerbil, Ssniff, Soest, Germany) and water was provided ad libitum. (DOCX) [file pone.0307551.s001.docx]

**Supplements**

**Hand raising and Keeping Conditions**

Animals were taken from our breeding colony at the Research Institute of Wildlife Ecology, University of Veterinary Medicine Vienna (for details see Bieber, 2009). For our experiment it was mandatory that the dormice were not afraid of humans. Therefore, we hand raised animals (in 2021 n=30, 13 males, 17 females and 2022 n=20, 14 males, 6 females). Pups were removed from their mother when their eyes started to open (~21 days as described in König 1960). Litters were born between June to July in both years. After removal from their natal nest, pups were held in a small transport box (40x26x23 cm, AniOne, Krefeld, Germany) and fed every 2 hours with human milk formula (Bepa expert HA PRE, Nestlé, Vevey, Switzerland). Powder of intestine bacteria was added (Enteroferment, Richterpharma, Wells, Germany) to prevent diarrhoea. At 24 days of age, pups were fed with milk every 3 hours. Fruit mesh (Bio Hipp Hippis, Hipp, Sachseln, Switzerland) and porridge (Milchbrei, Milupa, Frankfurt, Germany) was offered. At 25 days, sunflower seeds and apples were introduced and milk was reduced. To monitor growth, animals were weighted daily (balance 3pm Dipse TP 500, Oldenburg, Germany) to the nearest of 0.1 g. As pups became more mobile, they were transferred to birdcages (66x66x155 cm, VidaXL, Venlo, Netherlands) and were offered branches, platforms and nest boxes. At 27 days, pups were marked individually with transponders (ISO-Transponder, Tierchip Dasmann, Tecklenburger Land, Germany). At 30 days of age, pups were transferred to outside enclosures (2x1x1 m) with fenced floor, where they were kept in groups of 10 individuals. Nest boxes and branches were available, rodent chow (Ssniff Gerbil, Ssniff, Soest, Germany) and water was provided *ad libitum*.
